# Is intravenous urogram no longer an imaging of choice for percutaneous nephrolithotomy?

**DOI:** 10.4103/0970-1591.65413

**Published:** 2010

**Authors:** Pallavi Aga, Rajesh Bansal

**Affiliations:** Department of Radiodiagnosis, C.S.M.M.U. (KGMU), Lucknow, Uttar Pradesh, India; 1 Department of Urology, SGPGIMS, Lucknow, Uttar Pradesh, India

Imaging of kidney is an important part of the overall management of patients with kidney stones. It helps in assessing stone burden and its location, determining pelvicalyceal (PCS) anatomy and planning the mode of therapy, e.g., percutaneous nephrolithotomy (PNL), shock wave lithotripsy (SWL) or laparoscopy. In the West where computerized tomography urogram (CTU) has almost replaced intravenous urography (IVU) in evaluating most of the urological diseases, IVU is still the most frequently performed imaging in developing countries.[1]

Should CTU scan replace IVU before performing PNL? With changing trends in urological practice, this viewpoint deserves to be analyzed critically.

There is a level 1a evidence to suggest that noncontrast CT scan (NCCT) is the imaging of choice for patients presenting with acute flank pain.[2] Do we have the similar kind of evidence to say that CT is the best modality to treat stones before PNL?

Pfister SA 2003 compared the diagnostic accuracy of NCCT with IVU with a special interest on economic impact, applied radiation dose and time savings in patients presenting with acute colic.[3] A total of 122 patients were randomized for NCCT (n = 59) or IVU (n = 63). NCCT was found to be a better alternative than IVU because it had a higher diagnostic accuracy and was more effective as well as faster than IVU. But the radiation dose applied for IVU was 3.3 mSv and that for NCCT was 6.5 mSv. Unlike IVU, NCCT did not give an idea about the function of the involved kidney for which a contrast-enhanced CT scan is to be obtained, which would further increase the radiation dose.

In a noncomparative study with CTU on only 10 patients, stone site, number and size could be evaluated correctly in kidneys with complex pelvicalyceal anatomy. Optimal site for placing the percutaneous track with an additional knowledge of an association of this track with the abdominal organs could be assessed.[4] But the mean (range) radiation dose for all patients was 5.71 (3.2-9.2) mSv. In the same study, authors have stipulated that with only post-contrast CT imaging the mean radiation dose could be reduced to 1.7 (1.4-2.3) mSv.

Access for PNL using conventional fluoroscopic guidance may carry an increased risk of damage to surrounding organs in patients with abnormal anatomical situations such as horseshoe kidney, obesity and bony deformity. CT urogram in such difficult clinical situations helps in understanding PCS anatomy and relationship of stones to proposted puncture site.[5] But how far the CTU will help in treating stones in normal circumstance needs to be studied further.

CT scan undoubtedly gives a detailed anatomical view of the surroundings of the kidney and any other information unrelated to the stone disease. But in real life practice, injury to the surrounding organs in fluoroscopic or ultrasonography-guided puncture following an IVU is negligible. In a retrospective study, only 5 of 154 patients (3%) evaluated required the help of CT scan, in situations where retro-renal colon was present in 2 patients (suspected on ultrasonography) and a severely distorted body habitus due to spinal dysraphism in 3 patients. Percutaneous access was achieved without complication in the remaining cases.[6]

For PNL the most important step is to select a proper calyx for making a track to remove the maximum stone bulk in a single sitting. Most of the urologists even after viewing the initial imaging use fluoroscopy or intraoperative ultrasonography to make a puncture and establish a track. Fluoroscopy or ultrasonography plays a major role in establishing a track for PNL. While using fluoroscopy for making a puncture, the most common approach adopted is to use “bull's eye” technique considering that posterior calyces are located medially on anteroposterior imaging. For most urologists a target calyx is visualized after assessing an IVU film, which later is correlated on the image obtained on fluoroscopy.[7,8]

Despite this there is a general perception that CTU is a better choice than IVU. There is only one study till date, which has compared 3D CTU with IVU to plan a percutaneous access for nephrolithotomy in stag-horn stones and that also did not demonstrate its advantage over the IVU.[9]

With advances in the technology for CTU, it is impressive to obtain 3D-reconstructed color pictures of the stones. Whether obtaining 3D CTU extrapolates to a better outcome in treating stag-horn stones is yet to be seen.[10] There is a need for a prospective study that could compare CTU scan with IVU in the same patient in regard to the number of puncture, difference in technical ease of carrying out the procedure and the clearance rate. This might provide us with a reasonable level of evidence to have a scientific answer to this issue of the best imaging modality for PNL.

In practice, the choice of the most appropriate diagnostic imaging before PNL has been an IVU but a perception that CT would be more useful in treating large stone bulk is not based on any evidence. As IVU is less expensive, widely available and gives less radiation than contrast-enhanced CT, it is premature to replace this modality with CTU for treating stone disease with PNL.
